# Adverse Report of the Local Advisory Committee

**Published:** 1921-02-19

**Authors:** 


### THE BRADFORD MUNICIPAL HOSPITAL SCHEME.

### Adverse Report of the Local Advisory Committee.

It will be remembered that Dr. Addison has expressed himself of being desirous to obtain local medical opinion on the Bradford scheme, especially as the experiment would "have a bearing on any action which may have to be taken for the country as a whole." As a result of this wish, a local medical advisory committee was set up to consider and report on the St. Luke's Hospital scheme. A meeting of the medical men of the city was held on the 10th inst., when the report of the committee, with one or two amendments, was adopted. The report is lengthy, and it is possible only to give some of the salient features in these columns, and these are contained in the following extracts :

### Cost and Scope of Proposed Scheme.

In Dr. Buchan's report the number of beds available at St. Luke's Hospital is 1,148.

The cost per bed per week at the Royal Infirmary is nearly £4.

The cost to the Tuberculosis Committee for a tuberculous patient at a sanatorium is £5 8s. 6d. per week.

The Bradford Municipal Maternity Hospital costs very considerably more than either of the above.

Calculating upon these data, the cost to the ratepayers of Bradford for the upkeep of St. Luke's Hospital would be at least £240,000 per annum. This sum means at least 16s. per head of the population of Bradford per annum.

The present scheme contemplates taking over work which is now being adequately performed by the existing voluntary hospitals, thus producing an unnecessary and extravagant overlapping.

This Committee, therefore, can only approve of the St. Luke's Hospital scheme in so far as it is necessary to complete the adequate provision for the institutional treatment of Bradford's sick.

With regard to beds for in-patients, there is unquestionably a present deficiency, and this Committee would welcome the prospect of this being made up by the provision of a limited number of beds for general medical and surgical cases at St. Luke's Hospital.

### Criticism of Proposed Accommodation.

The criticism of the number of beds set out in Dr. Buchan's scheme as being necessary at St. Luke's Hospital is based upon the following considerations :

(*a*) Sir Napier Burnett, who is the authority upon hospital matters, has estimated that three beds per 1,000 of population is the number required in an urban community; this would mean 900 beds for Bradford.

(*b*) Apart from St. Luke's Hospital, clinics, and special hospitals, Bradford has in the three voluntary hospitals over 300 beds.

(*c*) Thus the number of beds required at St. Luke's Hospital is only 600, whereas the scheme provides for 1,148. Of the latter, 826 are allocated to medical and surgical cases, a number which closely approximates the total given in the above estimate as being necessary for the general requirements of the city.

(*d*) The number of beds allotted to the treatment of venereal disease is 108, but the Ministry of Health has approved as adequate the accommodation and treatment now provided at the Royal Infirmary.

The question, therefore, comes to be whether the Health Committee intends to be content to supply only such accommodation and treatment as are at present not satisfactorily available; if so, by co-operating with the Voluntary Hospitals Boards there will be no need for the full scheme as outlined in Dr. Buchan's report.

### Special Departments.

Turning to the question of special departments, the Committee considers that since there is abundant accommodation for the treatment of eye, ear, and throat cases at the Royal Eye and Ear Hospital, it considers that it is unnecessary to open a new department at St. Luke's Hospital, but rather to arrange with the Board of the Royal Eye and Ear Hospital for the treatment of St. Luke's cases.

With regard to the suggested out-patient departments, the Committee considers that adequate provision for out-patient treatment is already made at the existing voluntary hospitals, and, therefore, it cannot see any justification for burdening the ratepayers with the cost of a new department of this kind at St. Luke's.

The Committee is of the opinion that an efficient *x*-ray department should be established at St. Luke's, and also that experienced anæsthetists should be appointed.

### Proposed Alternative Scheme.

It is the opinion of this Committee that the projected hospital at St. Luke's should not occupy an isolated position, but should be linked up with the existing voluntary hospitals of the city by means of some such arrangement as obtains between St. Mary's Hospital, London, and the Paddington Infirmary. The Committee understands that this scheme has been in existence for some time, and is giving satisfactory results.

In pursuance of such a scheme similar arrangements would be necessary in Bradford to those existing in the St. Mary's scheme, by which the work at Paddington Infirmary is carried out by the junior honorary staff of St. Mary's Hospital. This would necessitate the enlargement of the assistant staff at the Bradford Royal Infirmary and other voluntary institutions in the city. In this way the honorary assistant staffs of the voluntary hospitals would become salaried officers of St. Luke's Hospital until such time as they were promoted to the senior staff of their respective voluntary hospitals, when their connection with St. Luke's would cease.

### Administration.

With regard to administration, the Committee is of the opinion that a Hospital Board should be constituted at St. Luke's Hospital to deal with the interior professional relations of the hospital and hospital arrangements, and that this Hospital Board should consist of the visiting staff together with the superintendent of the hospital and two general practitioners appointed by the Advisory Medical Committee.

